# *In situ* TEM observation of alpha-particle induced annealing of radiation damage in Durango apatite

**DOI:** 10.1038/s41598-017-14379-9

**Published:** 2017-10-26

**Authors:** Weixing Li, Yahui Shen, Yueqing Zhou, Shuai Nan, Chien-Hung Chen, Rodney C. Ewing

**Affiliations:** 1 CAS Center for Excellence in Tibetan Plateau Earth Sciences, and Key Laboratory of Continental Collision and Plateau Uplift, Institute of Tibetan Plateau Research, Chinese Academy of Sciences, Beijing, 100101 China; 2 Department of Geological Sciences, Stanford University, Stanford, CA 94305-2115 USA

## Abstract

A major issue in thermochronology and U-Th-Pb dating is the effect of radiation damage, created by *α*-recoils from *α*-decay events, on the diffusion of radiogenic elements (*e.g*., He and Pb) in host mineral. Up until now, thermal events have been considered as the only source of energy for the recovery of radiation-damage. However, irradiation, such as from the *α*-particle of the *α*-decay event, can itself induce damage recovery. Quantification of radiation-induced recovery caused by *α*-particles during *α*-decay events has not been possible, as the recovery process at the atomic-scale has been difficult to observe. Here we present details of the dynamics of the amorphous-to-crystalline transition process during *α*-particle irradiations using *in situ* transmission electron microscopy (TEM) and consecutive ion-irradiations: 1 MeV Kr^2+^ (simulating *α*-recoil damage), followed by 400 keV He^+^ (simulating *α*-particle annealing). Upon the He^+^ irradiation, partial recrystallization of the original, fully-amorphous Durango apatite was clearly evident and quantified based on the gradual appearance of new crystalline domains in TEM images and new diffraction maxima in selected area electron diffraction patterns. Thus, *α*-particle induced annealing occurs and must be considered in models of *α*-decay event damage and its effect on the diffusion of radiogenic elements in geochronology and thermochronology.

## Introduction

**Figure 1:**
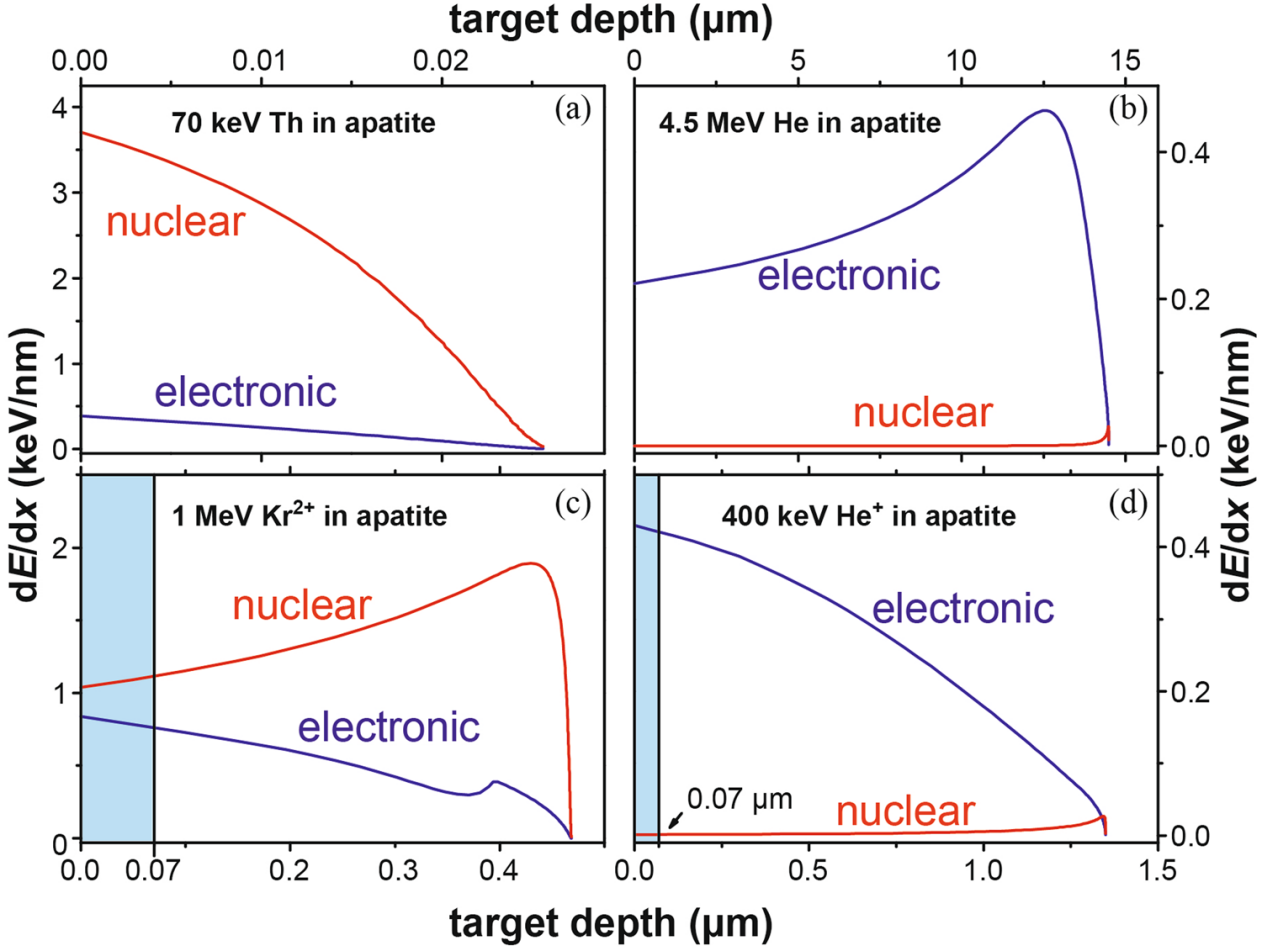
Stopping Power. The electronic and nuclear stopping powers, d*E*/d*x*, as a function of target depth, *x*, for the irradiations of 70 keV Th (*α*-recoil), 4.5 MeV He (*α*-particle), 1 MeV Kr (simulating *α*-recoil damage) and 400 keV He (simulating *α*-particle annealing), respectively. The TEM sample thickness (70 nm), for Kr and He ion-irradiation experiments, is marked by blue boxes.

Diffusion kinetics for noble gas thermochronometry is commonly assumed to be solely a function of temperature, and this is the basis for extrapolating the physical mechanisms observed in laboratory experiments to the temperature and time regimes of natural systems^1^. However, recent new models have demonstrated that alpha-recoil damage, *i.e*., isolated defects induced by α-recoils from α-decay events, significantly reduces the diffusion of noble gases (*e.g*., He) in apatite^1–4^. This effect can be reversed by thermal annealing of the radiation damage^2,3^. Similar approaches have been used in the determination of the age of the oldest zircon ~4.4 Ga *via* a new U-Th-Pb method^5–7^: The thermally enhanced Pb diffusion during a reheating event leads to the redistribution of the radiogenic ^207^Pb and ^206^Pb within the nano-clusters produced by alpha-decay. In addition, the recovery of fission tracks, another type of radiation damage caused by spontaneous fission of ^238^U, is generally considered as diffusion-controlled process^8–10^, an ultimate thermal effect. However, under certain radiation conditions, thermally induced diffusion is less significant than radiation-enhanced diffusion^11,12^ as more vacancies and interstitials are produced by interactions of energetic ions than those that are thermally activated^13,14^. In apatite, alpha-decay from U or Th produces a pair of an alpha-particle (energy: ~4.5 MeV He ion; ion range: ~14 µm), and an alpha-recoil (energy: 60–90 keV; ion range: 20–30 nm) that are ejected in opposite directions (Fig. 1a,b). This study addresses the recovery of alpha-recoil damage in apatite by the irradiation of alpha-particles, another source of energy that can drive the recovery process.

**Figure 2:**
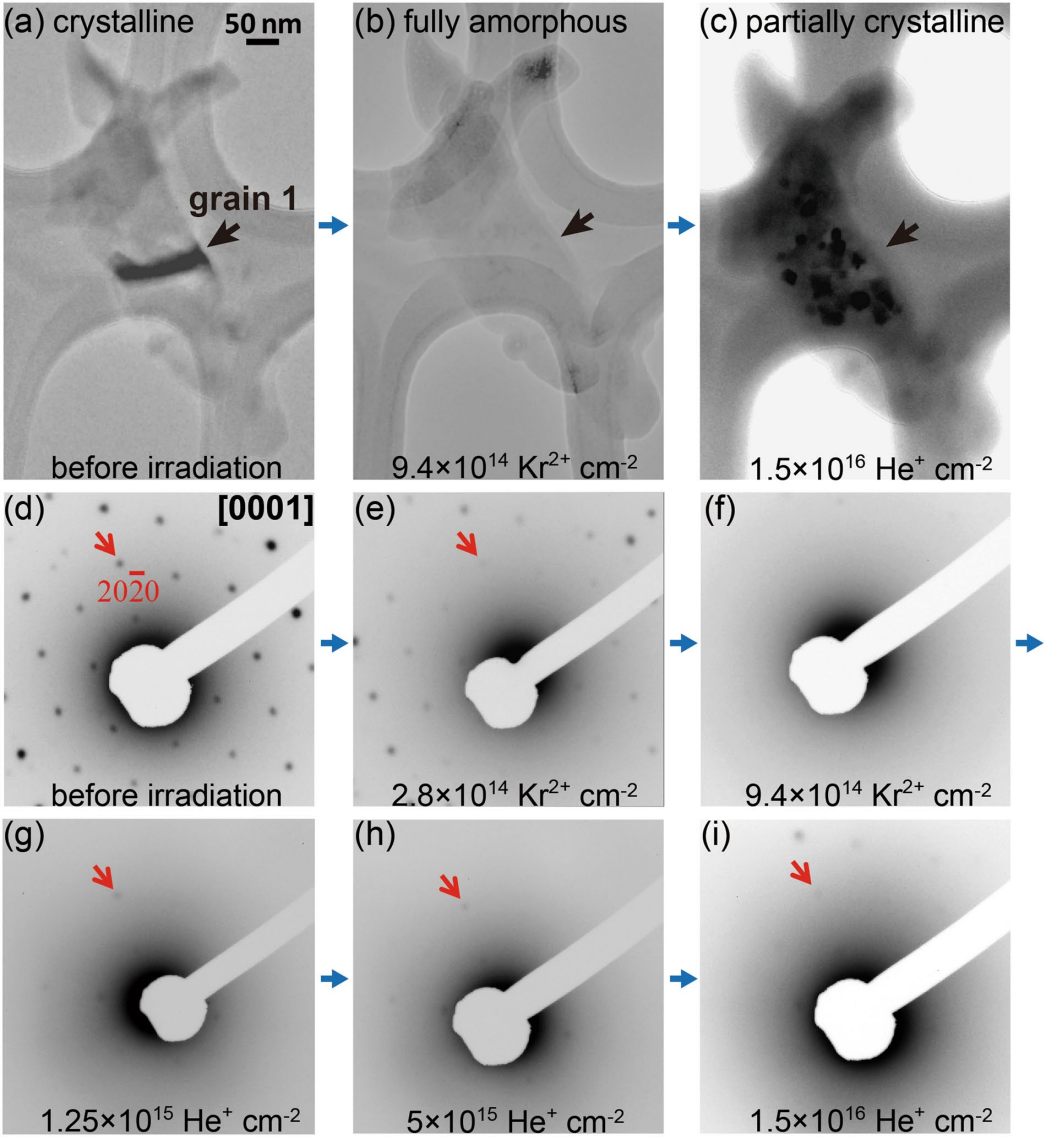
*In Situ* Experimental Results. *In situ* TEM images (upper row) and Selected Area Electron Diffraction (SAED) patterns (bottom two rows) from the same apatite (grain 1) setting on carbon thin film show (**a**,**b**) and (**d**–**f**), the gradual loss of crystallinity by Kr^2+^ ion irradiations (simulating *α*-recoil damage), and (**b**,**c**) and (**f**–**i**), the recovery of crystallinity by He^+^ ion irradiations (simulating *α*-particle recovery). The irradiation sequence (blue arrows), grain 1 (black arrows) and $(20\bar{2}0)$ (red arrows) are marked at different fluences to highlight the change during the two-step ion irradiations.

Despite the importance of radiation effects in reconstructing the thermal histories and age of rocks^1,5,10,15^, mechanisms of radiation damage and damage recovery in minerals are still poorly understood at the atomic-scale. Radiation damage in minerals is dominated by the accumulation of Frenkel defect pairs based on nuclear stopping power, (d*E*/d*x*)_*n*_, between heavy *α*-recoils (usually heavier than Pb) and surrounding atoms (Fig. 1a). This is in contrast to the irradiation of alpha-particles, where electronic stopping power, (d*E*/d*x*)_*e*_, between the alpha-particle and surrounding electrons, dominates over the entire ion range (Fig. 1b). Alpha-particles produce little damage by atomic collisions due to their light ion mass and low damage efficiency, *i.e*., nuclear to electronic stopping power ratio^16^. In certain materials, *e.g*., apatite and borosilicate glasses, preexisting radiation defects can be annealed by electronic excitation *via* electron-phonon coupling arising from the electronic energy loss^17–26^. Alpha-particles are more likely to cause the recovery of defects induced by alpha-recoils, than to create damage, because (1) the nuclear stopping power is negligibly low (~10^−4^ keV/nm) as compared with the electronic stopping power (0.2–0.4 keV/nm) (Fig. 1b), and (2) the electronic stopping power is significantly below the threshold (usually >10 keV/nm) for the creation of a nuclear ion track^27–29^, *e.g*., fission tracks, where the extremely high electronic energy loss is eventually transferred into atomic motion (or damage) *via* electron-phonon coupling^30^. A dual beam irradiation approach has been used to simulate the actinide alpha-decay events in a bulk curium doped glass^20^. In this study, we demonstrate how alpha-particles can cause the recovery of radiation damage in apatite using *in situ* TEM consecutive ion-irradiation experiments (see Method section). Careful control of the thickness and orientation of TEM specimens allowed us to acquire the nano- to atomic-scale information on microstructural change during the ion irradiations and to quantify the radiation-annealing power of alpha-particles from alpha-decay events by using a dose relation developed by this study.

## ***In situ*** Kr^2+^ ion irradiations

The morphologies and corresponding selected area electron diffraction (SAED) patterns of three sectioned slices (grain 1, 2 and 3), as prepared by the newly-developed microtome cutting method^9^, were recorded before and at each step during the interval of 1 MeV Kr^2+^ ion irradiations at room temperature (see details in Method). In the past three decades, 1 MeV Kr ions (or 0.012 MeV/nucleus) have been traditionally used in *in situ* experiments to simulate alpha-recoil (70 keV Th)^31–33^ because they both have a larger nuclear energy loss than electronic energy loss. All the sectioned slices have carefully-controlled sample thickness ~70 nm and crystallographic orientations close to the *c*-axis. As an example, the high crystallinity of grain 1 before irradiation is evidenced by significant phase contrast (*i.e*., the bright and dark contrast) in different regions of the grain, where a dark stripe is marked in the image (Fig. 2a). Such phase contrast in a crystalline specimen can be conveniently identified from low-magnification TEM images because any *small* changes in sample thickness, orientation or focus condition can alter the appearance of the image due to the differences in the phase of the electron waves scattered through the sample. The corresponding, hexagonal SAED pattern from the same grain 1 further confirms that this grain is crystalline, and the zone axis is oriented along [0001] (Fig. 2d). The product of *R*.*d* is constant, where *R* is the distance between (0000) and a diffraction spot in SAED patterns, and *d* is its corresponding lattice spacing. The intensity of diffraction maxima in the SAED diagrams has been difficult to quantify. For simplicity, only the number of diffraction maxima that are confined within the third-order hexagon is counted at each interval in order to quantify the radiation damage. Six equivalent diffraction maxima with the smallest *R* $(10\bar{1}0)$ = 0.12 Å^−1^, or the largest *d* $(10\bar{1}0)$ = 8.1 Å, form the first-order hexagon in grain 1. Twelve maxima $(20\bar{2}0)$ form the second-order hexagon, and eighteen outer ones form the third-order hexagon $(30\bar{3}0)$. Thus, the total number of diffraction maxima confined within the third-order hexagon [*i.e*., *R* ≤ 0.369 Å^−1^ (or *d* ≥ 2.7 Å)] is 36. The errors in counting the number of diffraction maxima can be minimized by the controlled sample orientation and uniform sample thickness.

**Figure 3:**
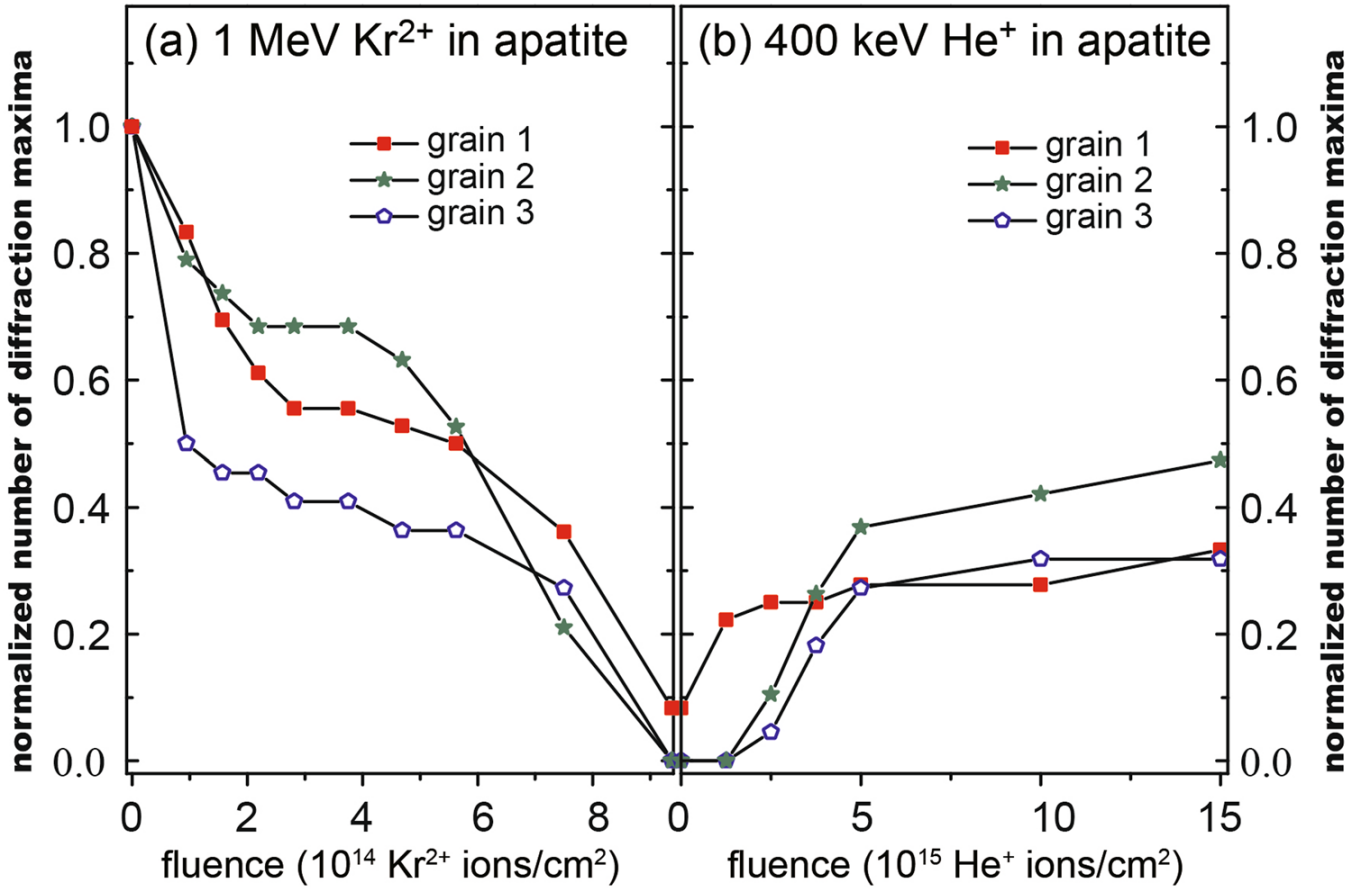
Quantification. Normalized number of diffraction maxima as a function of (**a**) Kr fluences and (**b**) subsequent He fluences. They are normalized by the original numbers of diffraction maxima, *i.e*., 36 maxima for grain 1 (red square), 22 for grain 2 (green star), and 19 for grain 3 (blue pentagon) before ion irradiations. The controlled sample thickness and orientation allow one to quantify the amount of radiation damage and He-induced damage recovery by counting the number of diffraction maxima from the SAED patterns for each grain.

**Figure 4:**
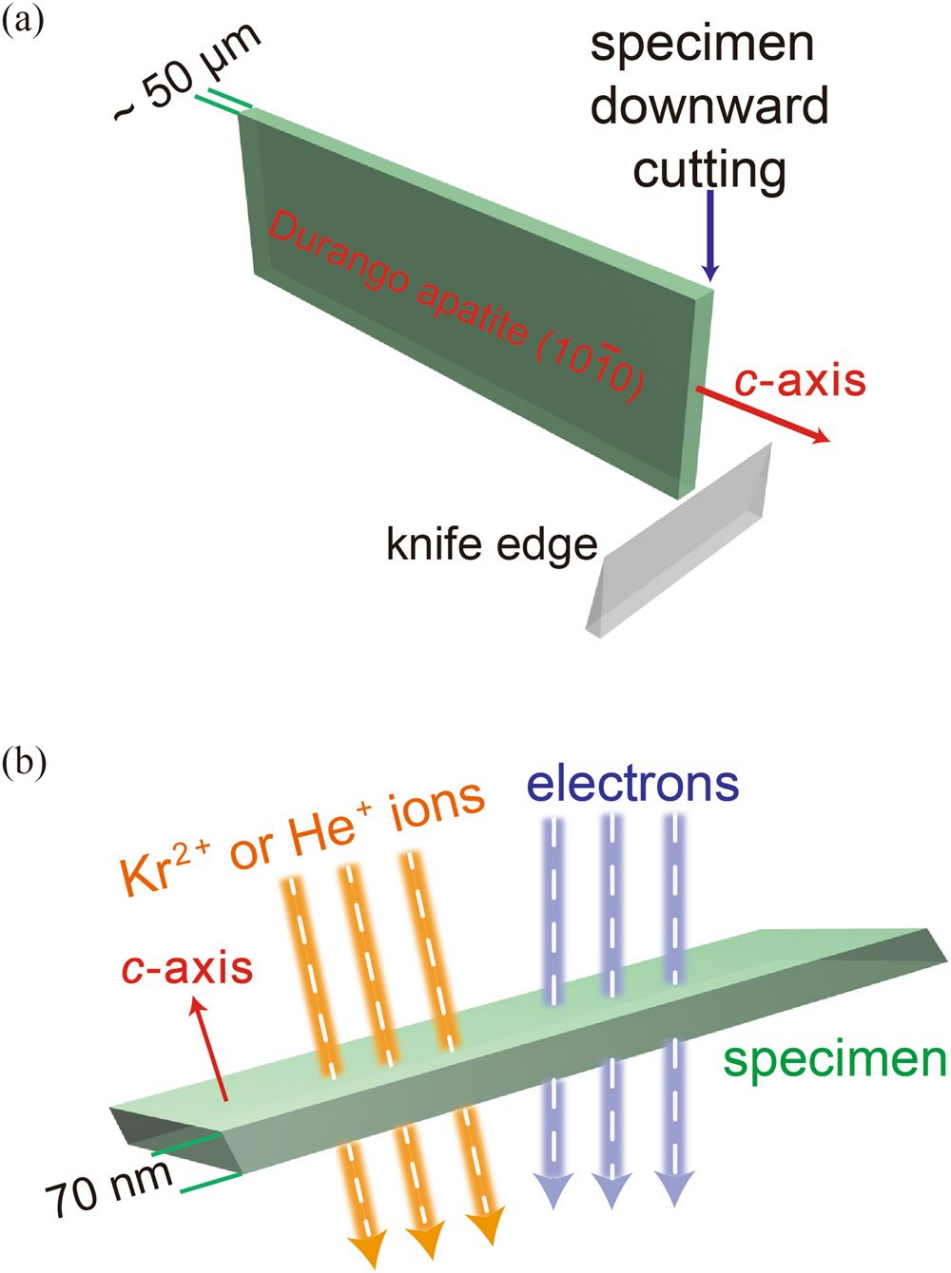
Experimental Methods. The microtome method (**a**) ensures all the sectioned slices with the same thickness (~70 nm) and orientation (along *c*-axis) for (**b**) *in situ* sequential ion irradiations and TEM observations.

Upon the irradiation of Kr^2+^, the gradual amorphization (or loss of crystallinity) induced by nuclear collision between incident ion and surrounding atoms is evidenced by the gradual disappearance of diffraction maxima (Fig. 2e). Interestingly, we found that the diffraction maxima corresponding to the first and second hexagons with larger *d* preferentially disappeared, indicating that the incident ion has a strong tendency to displace those atoms on the planes with large *d* values, up to 8.1 Å, *i.e*., *d* $(10\bar{1}0)$ in apatite. Other grains are also oriented along or very close to [0001], showing similar behavior under irradiation to grain 1 (not shown). The normalized number of diffraction maxima is plotted as a function of Kr fluence (Fig. 3a) and subsequent He fluence (Fig. 3b). Some grains have less diffraction maxima since some diffraction maxima are not apparent when the zone axes are not perfectly aligned to *c*. Notice that it is not technically feasible to meticulously tilt each grain perfectly parallel to the *c*-axis at each interval of the *in situ* TEM ion irradiation because at each fluence, the specimen is required to be orientated perpendicular to ion beam during ion irradiation, and then perpendicular to electron beam direction during observation (refer to Method and Fig. 4). The normalized numbers of diffraction maxima for the three grains initially drop rapidly from 1 to 0.5–0.7 around 2 × 10^14^ Kr^2+^ ion/cm^2^, followed by a steady transition stage with very slow decrease in the normalized number between 2 × 10^14^ and 6 × 10^14^ Kr^2+^ ion/cm^2^ (Fig. 3a). This process is followed by another accelerated decrease in the number of diffraction maxima until all the maxima are almost absent around 9 × 10^14^ Kr^2+^ ion/cm^2^ (Fig. 2f). However, some weak diffraction maxima still exist in the third order hexagon of grain 1, indicating the existence of a tiny amount of crystalline material and short range periodicity. As can be seen in the bright field TEM image (Fig. 2b), the almost complete amorphization of the Kr-irradiated sample can be further confirmed by the typical features of amorphous materials, such as the absence of phase contrast. Amorphous materials usually do not show such phase contrast as the aperiodic array of atoms scatter electron waves in random directions. As compared with the large variations for the conventional crushed powders, the three grains show a consistent, gradual disappearance of diffraction maxima due to the fact that the sample thickness and orientation are carefully controlled.

## ***In situ*** He^+^ ion irradiations

In order to simulate possible alpha-particle induced annealing effects, the Kr^2+^ pre-damaged apatite were further irradiated by 400 keV He^+^ ions at room temperature. Before the He irradiations, grain 1 was almost fully-damaged after Kr ion irradiations (Fig. 2b). Upon He ion irradiations, partial crystallinity is evident as can be determined by the bright and white phase contrast, resulting from the newly-formed nano-crystals (Fig. 2c). New nano-crystals can also be found in other grains including grain 2 and 3 (not shown). As clearly shown in Fig. 2c, these nano-crystals have strong tendency to form in certain areas. In other regions of grain 1, variations in phase contrast are not evident, and the regions are still amorphous. This indicates that the nucleation starts with a small, still crystalline domain that did not become fully amorphous during the Kr irradiation. Although the amorphous regions received the same amount of alpha-particle fluence, the recrystallization appears not to be initiated in the absence of a seed nucleus.

In addition to TEM images, further evidence of alpha-annealing can be seen from the changing sequence of diffraction patterns. For the lowest fluence of 400 keV He^+^ ions irradiations (1.25 × 10^15^ ion/cm^2^), the formation of new diffraction maxima is clearly evident in first and second diffraction hexagons in grain 1 (Fig. 2g–i), where these inner diffraction maxima originally disappeared after being irradiated to the fluence of 9 × 10^14^ Kr^2+^ ion/cm^2^. For grain 2 and 3, at or below 1.25 × 10^15^ He^+^ ion/cm^2^, the new diffraction maxima are too weak to identify (Fig. 3b), and formation of new diffraction maxima becomes evident when the fluence reaches 2.5 × 10^15^ He^+^ ion/cm^2^. As the He^+^ ion fluence increases, more new diffraction maxima appear, and the total number of diffraction maxima increases. Although the intensities of these diffraction maxima gradually increase, they are still difficult to quantify. The preferential formation of inner diffraction maxima (with larger *d* values) over outer maxima can be ascertained from the fact that new diffraction maxima first appear in the inner hexagons, followed by the appearance of diffraction maxima. Interestingly, it is clearly shown in the patterns that the locations of the new diffraction maxima during alpha-particle irradiations match exactly the previous locations of diffraction maxima before Kr ion irradiations. This indicates that under alpha-particle irradiations, the damaged apatite forms nano-crystals that are in the same orientation as the original structure of apatite, preferentially recovering the atom positions with larger *d*. Consistent with the nanocrystals observed in the TEM images, the diffraction patterns suggest that after Kr irradiations, the existence of crystalline seed nuclei allows for the initiation of recrystallization of the original structure. Full amorphization of a material has been routinely determined by the disappearance of all the diffraction maxima by TEM in the previous studies^34,35^. Thus, the grains are considered to be amorphous when all the diffraction spots disappear after Kr ion irradiations. However, because of the recrystallization along exactly the same orientation, it is likely that there is a glass-like material where the aperiodic rotations of phosphate tetrahedra are sufficient to distort the atoms and cause the disappearance of diffraction spots after Kr ion damage, while the fluorine channels of the fluorapatite structure remained almost intact, as discovered by the Molecular Dynamics (MD) simulation^36^. The undamaged fluorine channels^36^ after Kr ion irradiation may provide a framework for the recovery of the structure in the same orientation by the irradiation of alpha-particles.

During He irradiations, the number of diffraction maxima as normalized by the initial number of diffraction maxima of the un-irradiated samples increase from ~0 before He irradiation to 30–40% as the fluence reaches 5 × 10^15^ He^+^ ion/cm^2^. Above this fluence, a steady-state stage follows for which the rate of damage production is approximately equal to the rate of annealing, as very few new diffraction maxima appear. At the highest fluence (1.5 × 10^16^ He^+^ ion/cm^2^), the number of diffraction maxima induced by the He irradiations is close to 40% of the initial number of diffraction maxima for the un-irradiated samples.

## Quantification of the dpa in natural apatite from ***in situ*** Kr ion irradiations

Radiation damage is conventionally quantified using displacements per atom (dpa) that result from nuclear collisions^35,37^. However, the random orientation of crushed samples makes it difficult to quantify the amount of radiation damage, as it is hard to tilt all the samples, each with a different orientation, to align along a low-indexed zone axis during *in situ* TEM ion irradiation experiments. In contrast, in this study all the sectioned slices of apatite are oriented along the same major zone axis [0001], which allowed us to easily monitor the microstructural changes of each sectioned slice, and to quantify radiation damage by counting the diffraction maxima from the well-defined, hexagonal regions in the SAED patterns of apatite along *c*.

Natural apatite does not become fully metamict^10^. This is in contrast to frequently occurring, metamict zircon, which contains up to 4,000 ppm uranium and up to 2,000 ppm thorium^38^. The absence of the fully-metamict state in natural apatite has been attributed to the inability of apatite to accumulate radiation damage from alpha-decay^10^, but details of this process have not been elucidated. When a specimen is heated above a critical amorphization temperature, *T*_*c*_ (apatite: 475 K *v.s*. zircon: up to 1,000 K)^39^, a measure of the susceptibility of a material to radiation damage as investigated by *in situ* ion irradiations, it cannot be damaged to the point that it is fully amorphous. Apatite is more likely to remain crystalline at the same geological environment (*e.g*., temperature) because it has a lower *T*_*c*_ than zircon. On the other hand, the radiation-annealing effects of alpha-particles, as investigated by He ion irradiations using Rutherford Backscattering Spectrometry (RBS), have been used to explain why metamict apatite is never found in nature^23^. However, quantification of radiation-induced recovery has been difficult because this process is caused by electronic interactions, which are different from atomic displacements and cannot be quantified by dpa. In this study, the loss and recovery of diffraction maxima from the low-indexed zone axis of [0001] during the *in situ* TEM ion irradiations by using the specimens with controlled orientation and thickness provide a great opportunity to quantify the radiation-annealing power of naturally-occurring alpha-particles.

According to Monte Carlo simulation of SRIM program^40^, a 1 MeV Kr^2+^ ion produces an average of ${n}_{{\rm{Kr}}}$ = 0.83 vacancies (or displacements) per unit depth per ion, in ${\rm{v}}{\rm{a}}{\rm{c}}{\rm{a}}{\rm{n}}{\rm{c}}{\rm{i}}{\rm{e}}{\rm{s}}\,/({\rm{i}}{\rm{o}}{\rm{n}}\,.\AA )$, in fluorapatite, throughout the entire thickness of sectioned slices ~70 nm. Therefore, the critical amorphization dose, is the point where all diffraction maxima disappear at *D*_*kr*_ = 9 × 10^14^ Kr^2+^ ion/cm^2^ (Fig. 2a), and can be converted to a dose in dpa by,

1 $${D}_{{\rm{dpa}}}=\,\frac{{10}^{8}.{n}_{{\rm{Kr}}}.{D}_{{\rm{Kr}}}\,}{m}=0.93\,({\rm{dpa}}),\,$$

where the atomic number in a unit volume, *m* = 8.0 × 10^22^ atoms/cm^3^ can be obtained from the density, *ρ* = 3.2 g/cm^3^. The dpa ~0.93, as obtained in this study, is significantly larger than the widely cited, the critical amorphization dose ~0.25 dpa, as was measured by 1.5 MeV Kr^2+^ in fluorapatite at 273 K from crushed powders without controlling for sample thickness and orientation^39^. In fact, a 1.5 MeV Kr^2+^ ion and a 1 MeV Kr^2+^ ion do not show much of a difference in production of dpa, because their ${n}_{{\rm{Kr}}}$ values are close, *i.e*., 0.7 for 1.5 MeV Kr^2+^ *vs*. 0.83 ${\rm{v}}{\rm{a}}{\rm{c}}{\rm{a}}{\rm{n}}{\rm{c}}{\rm{i}}{\rm{e}}{\rm{s}}\,/({\rm{i}}{\rm{o}}{\rm{n}}\,.\AA )$ for 1.0 MeV Kr^2+^, respectively. In order to confirm the measured dose in this study, we measured other grains (>9 grains) where the critical amorphization doses are consistently about 0.93 dpa for each. However, the dose (0.21 dpa) at which the diffraction maxima change from rapid disappearance to steady disappearance (Fig. 3a), is very close to the previously measured critical amorphization dose^39^. The significantly larger critical amorphization dose, as determined in this study, indicates that apatite is more resistant to radiation damage than was previously thought, and this explains why apatite in nature is never fully-amorphous. However, according to previous results, apatite has a lower critical amorphization dose than that of zircon ~0.55 dpa at room temperature^41^. Thus, the difference in the critical amorphization dose has not been used to explain the absence of fully metamict state in natural apatite^10^. More importantly, the immobilization timescale for apatite as a nuclear waste form for the incorporation of radionuclides^42^ is significantly enhanced because of the three times greater amorphization dose than had been previously estimated^39^.

The concentrations of U and Th in natural apatite are typically less than 50 ppm U^10^. In Lake Mountain apatite, due to its unusually high concentration of actinides (U: 146 ppm; Th: 3 ppm; He age: 329 Ma)^1^, the alpha-dose, *D*_*α*_ = 1.6 × 10^17^ alpha-decays/g, can be calculated as mentioned elsewhere^38,43^. This alpha-dose can be converted to the dose in dpa by,

2 $${D}_{{\rm{dpa}}}=\frac{{n}_{\alpha }.{D}_{\alpha }.\rho \,}{m}=4.6\times {10}^{-3}\,({\rm{dpa}}),$$

where the total vacancies produced by an alpha-recoil (*e.g*., 70 keV Th) during an alpha-decay event, ${n}_{\alpha }=715\,\mathrm{vacancies}\,/\alpha$, along its entire path around 25 nm, can be obtained using SRIM program^40^. The number of vacancies for alpha-recoil has been estimated to be 1,000~2,000 in zircon and apatite^41,44,45^. However, more recent work reported much higher values, ~5,000 for alpha-recoil in natural zircon^46^ and 10,000 for ^239^Pu in a PuCoGa_5_ superconductor^47^, using Nuclear Magnetic Resonance (NMR) and Extended X-Ray Absorption Fine Structure (EXAFS), respectively. The MD simulation gives a final ~2,000 displaced atoms per 5 keV recoil in apatite^36^. In order to compare 70 keV Th damage in natural apatite and 1 MeV Kr ion irradiations, SRIM simulation is used in both cases for consistency. This is because the modern experimental and simulation results on displaced atoms for a 1 MeV Kr (~200 times larger energy than a 5 keV recoil) in apatite are not available, and they are difficult to estimate. However, more accurate dose relation can be made in the future when the experimental or MD simulation results for both alpha-recoil and 1 MeV Kr are available. As alpha-recoil induced cascades are randomly oriented, the alpha-dose has a unit of alpha-decays/g, or alpha-decays/cm^3^, in contrast to the ion fluence (in ion/cm^2^) for parallel ions during ion irradiations. However, the use of dpa allows one to conveniently compare the amount of damage in apatite for these two different ions. The typical values from natural apatite, *e.g*., 1.5×10^−4^ dpa in Durango apatite (U: 8 ppm, Th: 180 ppm and age: 31 Ma)^1^, are several orders of magnitude less than the full amorphization dose ~0.93 dpa, as determined in this study. According to the “trapping” model^1^, the effect of radiation damage on He diffusion is quite significant, corresponding to tens of degrees variations in closure temperatures across the range of typical apatite eU ([U] + 0.235[Th]) around 4–150 ppm. Although the dpa for a typical apatite is ~3 orders of magnitudes less than the full amorphization dose, the level of defects of natural apatite is high enough to significantly hamper He diffusion by trapping He in isolated defects^1–4^. In addition, alpha-particle induced annealing causes significant recovery of the radiation damage from alpha-recoils, which makes natural apatite much more difficult to amorphize.

## Quantification of ***α***-annealing effects on natural apatite by ***in situ*** ion irradiations

Despite the much higher energy (4.5 MeV) of an alpha-particle than that of a typical alpha-recoil (*e.g*., 70 keV Th), only a total of ~130 vacancies can be produced, mainly located at the very end of the ion path due to the lower damage efficiency of the lighter element He. This is because heavy elements have a much greater damage efficiency than light elements for producing radiation damage induced by nuclear collisions^16^. The electronic excitation arising from electronic interactions of the intermediate-energy heavy-ions (*e.g*., 21 MeV Ni ions) in ion irradiation experiments has been found to heal pre-existing radiation damage^21,25^. As compared with the intermediate-energy heavy-ions, the lighter, less energetic alpha-particles are more likely to lose energy by electronic interactions, and they are more efficient in triggering the recovery of the radiation damage. Recovery of extended defects during helium-ion irradiations, can be attributed to radiation-enhanced diffusion^14^, an increase in defect-mobility caused by electronic interactions.

The ion irradiation experiment provides important information on the annealing effects of the *α*-particles on natural apatite. However, quantification of the annealing effects of the 4.5 MeV *α*-particles from actinide decay using the experimental results of 400 keV He ion irradiations is not as conveniently done as the quantification of the amount of damage for the two heavy ions (1 MeV Kr, and 70 keV Th) using dpa. There are two factors that prevent the direct comparison of the annealing effects of the two different ions: (1) The effective length (or the penetration lengths ~14 µm) of alpha-particles in a bulk sample is much longer than that in ion irradiation experiments where the 400 keV He-ions penetrate the entire TEM sample thickness ~70 nm; (2) The dose of randomly oriented alpha-particles is quantified by alpha-dose in a unit of volume (*α*-events/cm^3^) in natural apatite, while the fluence of the parallel He ions is quantified in a unit of area (in ions/cm^2^). In order to calculate He ion affected area, we assume that, similar to the case of fission track formation in apatite^48^, the area (π*R* ^2^, where *R* is the radius) affected by a He ion is proportional to electronic stopping power,

3 $$S=\pi {R}^{2}=k.{({\rm{d}}E/{\rm{d}}x)}_{e},\,k\,{\rm{is}}\,\,{\rm{a}}\,{\rm{constant}}.$$

Thus, the affected volume by an individual He ion can be defined by the product of area affected along the ion, and the penetration length of the ion. For the randomly oriented 4.5 MeV *α*-particles in natural apatite, the ratio of the He-ion affected volume in a bulk sample is the product of the average affected volume along an individual, randomly-orientated alpha-particle, $\overline{{S}_{b}}.{L}_{b}$(in cm^3^), and the alpha-dose, $b\,$(in *α*-events/cm^3^), where $\overline{{S}_{b}}$ is the average affected area, and ${L}_{b}$ the penetration length ~14 µm. Similarly, for the parallel 400 keV He ions penetrating a TEM sample, the ratio of the average affected area, $\overline{{S}_{a}}$ (in cm^2^), in a TEM sample is the product of affected area along an individual, parallel He ion and the ion fluence of $a$ (in He-ions/cm^2^). In order to reach the same annealing effects in the two conditions, we assume that the two ratios must be equal, that is,

4 $$(\frac{{\int }_{0}^{{L}_{b}}{S}_{b}dx}{{\int }_{0}^{{L}_{b}}dx}.{L}_{b}).b=\frac{{\int }_{0}^{{L}_{a}}{S}_{a}dx}{{\int }_{0}^{{L}_{a}}dx}.a,$$

where, ${L}_{a}$ ~70 nm as the 400 keV He ions penetrate the entire TEM sample thickness. From Equations 3 and 4, and Fig. 1b,d, we obtain the dose relation between the irradiations of alpha-decay in nature and the He ion-irradiation simulations in this study,

5 $$a\cong 0.7{L}_{b}b,\,\mathrm{in}\,\mathrm{ions}/{{\rm{cm}}}^{2}.\,$$

A direct quantification of alpha-particle annealing effects on alpha-recoil damage is technically difficult despite its importance to both thermochronology and nuclear waste form development. The dose relation above provides an opportunity to quantify the alpha-particle annealing effect induced by alpha-decay based on the results of *in situ* TEM ion irradiations. In a high alpha-dose case, the change in the normalized numbers of maxima is large enough for the quantification of the degree of radiation damage from alpha-recoils as simulated by 1 MeV Kr and that of damage recovery from alpha-particles as simulated by 400 keV He. For example, as a nuclear waste form, apatite contains up to 5 wt% Pu, and the alpha dose in 1,000 years reaches up to 1 × 10^19^ alpha-events/cm^3^ ^41^. As can be calculated by Equation 5, the annealing effect of alpha-particles from alpha decay events is equivalent to that of ~1 × 10^16^ 400 keV He ions/cm^2^. This corresponds to an average increase (+30%) in the normalized number of diffraction maxima from 0% based on grain 1, 2 and 3 (refer to Fig. 3). On the other hand, as an alpha-decay event produces an alpha-particle and an alpha-recoil, the amount of damage of alpha-recoils (1 × 10^19^ alpha-recoils/cm^3^) in the same material is equivalent to that of 9.4 × 10^13^ 1 MeV Kr^2+^ ions in the ion-irradiations (refer to Equations 1 and 2). This corresponds to a drop (−30%) of the normalized number of diffraction maxima from 100% to an average of 70% based on grains 1, 2 and 3 (refer to Fig. 3). Through a direct comparison of the change of the normalized number of diffraction maxima in the damage production process and alpha-annealing process, we conclude that the recovery effects of alpha-particles on radiation alpha-recoil induced damage are significant. More importantly, there is near balance as indicated by the steady-state stage during the irradiations by alpha-particles.

In natural apatite, the defect level produced by alpha-recoils is significantly lower than that is expected in apatite used to incorporate actinides, such as plutonium. In Lake Mountain apatite, the annealing effect of 5.1 × 10^17^ 4.5 MeV alpha-particles/cm^3^ equals to that of 5.1 × 10^14^ 400 keV He ions/cm^2^ in the ion-irradiation experiments according to the above dose relation. The corresponding alpha-recoils (5.1 × 10^17^ 70 keV Th ions.cm^−3^) produce a dpa equivalent to the dpa produced by 4.8 × 10^12^ 1 MeV Kr^2+^ ions/cm^2^ by ion irradiations (refer to Equation 1). However, a direct quantification of radiation damage and damage recovery by the normalized numbers of maxima at such low doses is difficult. The changes in the numbers of diffraction maxima for both 4.8 × 10^12^ 1 MeV Kr^2+^ and 5.1 × 10^14^ 400 keV He ions.cm^−2^ are less than 10%, which are difficult to identify precisely from Fig. 3. However, it is clearly shown in Fig. 3b that the annealing effect of 400 keV He is more significant at low dose than at high dose. Thus, it is reasonable to assume that the annealing effect of alpha-particles in naturally-occurring apatite is as significant as (if not more significant) at low doses as compared with high doses. In addition, the doses of alpha recoils in natural apatite are high enough to significantly hinder the diffusion of He, as demonstrated by the “trapping” model, even at low doses^1^. Thus, the recovery of alpha-recoil damage that is induced by alpha-particles should be considered in the “trapping” model of He diffusion in a radiation damaged mineral.

In summary, the partial recrystallization of the original, fully-amorphous Durango apatite (pre-damaged by 1 MeV Kr^2+^) was observed by *in situ* ion irradiations with the gradual appearance of new crystalline domains (from TEM images) and new diffraction maxima (from SAED patterns) under the irradiation of 400 keV He^+^. The room-temperature critical amorphization dose for apatite, as determined in experiments in which sample thickness and crystallographic orientation are carefully controlled is three times greater than that determined on powdered samples of apatite^39^. The alpha-particle induced recrystallization back to the original structure appears to be initiated from very small, undamaged domains that did not become amorphous by Kr ion irradiation, as details of this process at the nano- to atomic-scale were observed in the *in situ* TEM experiments. Quantification of radiation damage and damage recovery was achieved by using a dose relation derived through the change in the number of diffraction maxima in TEM specimens with a controlled thickness and orientation. Although the thermal annealing of radiation damage in apatite has been studied in detail, our results emphasize the importance of considering the effect of radiation-induced annealing, particularly by *α*-particles, in apatite. The final atomic-scale structure of a damaged material will be determined by the amount of damage created and the degree of recovery caused by both thermal and irradiation events.

## Method

The specimens for *in situ* ion irradiations were prepared by a newly-developed microtome cutting method^9^ to ensure all the samples with uniform sample thickness (~70 nm) and identical crystallographic orientation, *i.e*., the *c*-axis of fluorapatite [Ca_5_(PO_4_)_3_F] from Durango, Mexico (Fig. 4a). Apatite was annealed at 500 °C for 5 h to totally remove the pre-existing, naturally-occurring radiation damage. Specimen was cut into slices 200–500 μm thick by a diamond saw along with the *c-*axis, allowing the cut surface parallel to $(10\bar{1}0)$. The cut slice with the marked *c-*axis was further thinned down to a thickness ~50 μm by polishing both sides of $(10\bar{1}0)$. The polished specimen was mounted in an Ultracut E microtome with the polished surface glued onto a flat supporting material (Fig. 4a). The marked *c-*axis was oriented perpendicular to the diamond knife edge so that after the microtome cut, the zone axis of all the sectioned slices is along the *c*-axis. The thickness of sections was controlled by the automatic advance of the mounted specimen, ~70 nm for each cut. The sectioned slices were floated onto distilled water before being loaded onto a carbon thin film TEM grid. However, under TEM observation, a perfect zone axis after tilting for a specific slice does not stand for other slices due to possible bending of the supporting carbon thin film under each slice.

For *in situ* TEM ion irradiations, specimens should be thin enough (usually <100 nm) to allow both electron-beams and ion-beams to penetrate through the entire sample. At least 3–5 well-separated fine specimens are required to avoid the uncertainty in the determination of the amount of damage. A cross-sectional sample is not often used due to the limited sample number. Instead, specimens are usually prepared by dispersing crushed fine powders of crystals on a holey carbon TEM grid to obtain numerous grains. However, crushed fine powders vary in sample thickness, which causes large statistical errors in the determination of the amount of damage^35,39^. More importantly, it is more difficult to monitor the atomic-scale structural change by *random* diffraction spots of the SAED patterns from the crushed powders without controlling sample orientation than *ordered* diffraction spots from a major zone axis, *e.g*., [0001] (see Fig. 2). Therefore, a sample with the controlled orientation and sample thickness, as prepared by microtome cutting, is greatly favored when doing *in situ* TEM ion irradiations and minimizing the statistical errors.

The microstructural change of crystalline-to-amorphous transition during the 1 MeV Kr^2+^ ion irradiations and subsequent damage recovery of apatite during the 400 keV He^+^ ion irradiations was directly recorded at room temperature by *in situ* TEM connected to the beam line of the IVEM Tandem Facility at the Argonne National Laboratory (Fig. 4b). The ion-beam enters the TEM column having a uniform irradiated area on the sample of a 2 mm diameter circle in the centre of a standard 3 mm TEM grid. The ion beam is incident at 30° from the microscope optic axis and electron beam. This setting assures that the ion beam is perpendicular to the specimen during ion irradiations. At the same time, this allows for imaging the morphologies and the SAED patterns of the specimen in the interval of ion irradiations by sample tilting, that is, tilting 30° back to make the *c*-axis of apatite parallel to the electron beams. In this way, the two-step irradiations can be monitored by observing gradual change in both SAED pattern and corresponding morphology of a specimen as a function of irradiation fluence (Fig. 2). The critical amorphization dose, *i.e*., the fluence when the specimen is fully amorphous, is determined as the fluence where all the diffraction maxima are no longer evident. Ion dosimetry is monitored using Faraday cups within the microscope and 2 cm from the sample. The temperature rise of the target materials during the ion (or electron) irradiations is primarily proportional to flux or current density^49,50^. The flux for the ion irradiations of He^+^ or Kr^2+^ was 6.25 × 10^11^ ions/cm^2^/s or less. This flux is low enough to ensure that the temperature variations as measured by the temperature controllers were no more than 2 K^51^. During the TEM observations, the current density is kept as low as possible (0.1–1 A/cm^2^, or around 3 × 10^18^ e^−^/cm^2^/s). It has been reported that the temperature rise by electron beams is ~10 K or less, which is not high enough for the thermally-induced recrystallization^52^.

Actually, a typical alpha-recoil, such as 70 keV Th ions, is not suitable for *in situ* TEM ion irradiations because complete amorphization cannot be achieved in a TEM specimen due to an insufficient ion range (~20 nm) to penetrate the entire thickness of the specimen 50–200 nm (Fig. 1a,c). In the last two decades, the low-energy (below 1 MeV/nucleon) heavy-ions, *e.g*., 1 or 1.5 MeV Kr^2+^ ions, have been routinely used in *in situ* ion-irradiation experiments to obtain the critical amorphization dose caused by the alpha-recoils^35,37,39^. This is because these Kr^2+^ ions readily penetrate a TEM specimen, and more importantly the nuclear collisions of these ions dominate over the electronic interactions, which is comparable to that of alpha-recoils (Fig. 1c).
